# A lower dosage of imatinib in patients with gastrointestinal stromal tumors with toxicity of the treatment

**DOI:** 10.1097/MD.0000000000005488

**Published:** 2016-12-09

**Authors:** Yuan Yin, Jin Xiang, Sumin Tang, Jiaju Chen, Qin Yu, Bo Zhang

**Affiliations:** a Department of Gastrointestinal Surgery; b Laboratory of Clinical Pharmacology, West China Hospital, Sichuan University, Chengdu, Sichuan, China.

**Keywords:** adverse events, dose reduction, gastrointestinal stromal tumor, imatinib

## Abstract

This study investigated the efficiency and safety of imatinib in the lower dose (300 mg/d) in patients with gastrointestinal stromal tumor (GIST) who cannot tolerate imatinib in the standard dose (400 mg/d).

Steady-state imatinib trough concentration (Cmin) values in 18 patients with GIST who were taking 300 mg/d or 400 mg/d imatinib were measured. The clinical features, toxicity data, and follow-up data were collected.

Around 18 patients with GIST were investigated in which 9 patients received 300 mg/d imatinib. The mean imatinib Cmin value of the 18 patients was 1841 ng/mL (1018–3897 ng/mL). The difference between the patients treated with 400 mg/d (n=9) and those treated with 300 mg/d (n = 9), which have imatinib Cmin values of 2122±1003 ng/mL and 1559±478 ng/mL, respectively, was not significant (*P* = 0.148). In total, 12 of the 18 patients had complete resection of the primary tumor, 8 of whom received postoperative imatinib 300 mg/d. After the average follow-up of 15.4 months, no recurrence was documented. Of the 6 patients with unresected GIST, 1 received imatinib 300 mg/d for 13 months. The tumor size of this patient continued to decrease. In contrast to patients treated with imatinib 400 mg/d, patients treated with imatinib 300 mg/d notably exhibited lesser drug-related side effects.

Patients with GIST who exhibited intolerance to the standard dose of imatinib (400 mg/d), a lower dose of 300 mg/d could provide not only sufficient plasma Cmin and good disease control but also the alleviation of the side effects.

## 1 Introduction

Gastrointestinal stromal tumors (GIST) are the most common mesenchymal tumors of the alimentary tract, accounting for ∼1% to 3% of all malignant gastrointestinal neoplasms. Activating mutations of the KIT or platelet-derived growth factor receptor (PDGFRA) gene has been demonstrated to play a key role in tumorigenesis.^[1,2]^ Introduction of imatinib (IM), which is a tyrosine kinase inhibitor that targets protein kinases, into the treatment of GISTs has notably improved the clinical outcome of this disease. IM has become the first-line treatment of patients with advanced and unresectable GIST, and the primary drug in adjuvant treatment for GIST patients with high risk of recurrence who underwent surgical resection.^[3–6]^

PK studies have suggested a rapid and nearly complete (98%) oral bioavailability of IM and the elimination half-life of ∼20 hours, thus allowing once-daily dosing.^[7]^ According to the previous studies, maintaining the plasma trough concentration (Cmin) of IM at higher levels is associated with better clinical outcome for patients with advanced unresectable GISTs.^[8]^ The standard dose of IM is 400 mg/d. In order to achieve higher Cmin and better response, a higher dose of 800 mg/d or 600 mg/d is recommended for patients with kit 9 exon mutations or progressed on 400 mg/d.^[4,5]^ However, high-dose IM is associated with severe drug-related side effects in most cases. In some cases, even a standard dose of 400 mg/d is intolerable. Side effects such as fluid retention, diarrhea, nausea, rash, abnormal liver function test, and hematologic adverse reaction have been frequently reported. Although the side effects may improve with prolonged therapy and usually be managed by appropriate supportive measures, suspension, or reducing the dose of IM treatment is common in clinical practice.^[4,5]^ It is reported that the interruption of IM is associated with rapid progression,^[9]^ and NCCN guidelines for sarcoma recommends the continued use of IM treatment at a reduced dose of 300 mg/d if the side effect is mitigated after suspension.^[10]^ However, whether the lower dosage of IM is sufficient to maintain an ideal Cmin and therapeutic efficiency has been unclear. Information on Cmin and on the response in patients with GIST administered with reduced dose of IM because of severe or long-term side effect is insufficient.

## 2 Materials and methods

### 2.1 Patients

Eighteen patients with confirmed GIST and treated with IM between 2010 and January 2015 in West China Hospital, Sichuan, China, were identified. Twelve of these patients had undergone surgery. All patients were treated with 400 mg/d IM initially for at least 4 weeks. We excluded patients who had been treated with other tyrosine kinase inhibitors or other dose of IM. The dose was reduced to 300 mg/d in 9 patients because of severe or long-term low grade toxicity. Dose reduction was made cautiously and personally after closely monitored detailed discussion and instruction with the patient. The other 9 patients had been administered with 400 mg/d IM consistently since the start of treatment. IM Cmin was measured in January 2015. Each patient received a dose of IM regularly at 12 am daily for at least 4 months by the time of Cmin assessment. The participants provided written informed consent. Clinical data, including all the radiological images, were reviewed retrospectively. Body weight, BSA, complete blood counts, serum chemistry, and electrolytes were measured within 7 days of each PK assessment. Response to IM treatment was defined according to the Response Evaluation Criteria in Solid Tumor (RECIST).^[11]^ Toxicities were assessed according to the National Cancer Institute common toxicity criteria. The West China Hospital Research Ethics Committee approved the retrospective analysis of anonymous data involved in this study. Patient records were anonymized and de-identified prior to analysis.

### 2.2 PK analysis

Patients with serious comorbidity or who were administered with concomitant medications that could inhibit or induce cytochrome 3A enzymes were excluded. Four milliliters of blood samples were collected into heparinized tubes at 10 am, nearly 22 hours after the previous dose of IM. The collected blood was centrifuged at 3000 rpm for 10 minutes for plasma separation, and was kept at −20°C for analysis. The plasma concentrations of IM were measured through liquid chromatography tandem mass spectrometry assay in the clinical laboratories of West China Hospital.^[12]^ The IM Cmin in 5 patients was measured twice on different days, and then the mean of the 2 values were used for analysis. Meanwhile, the IM Cmin in the other patients was measured once and the resulting value was used for analysis.

### 2.3 Statistical analysis

The Wilcoxon rank test was used to compare the means of the IM Cmin of patients treated with different doses of IM. The value of IM Cmin was log-transformed for the linear regression analysis in order to identify the correlations between the IM Cmin and the covariates, such as age, sex, body weight, BSA, duration of IM use, hemoglobin, WBC, ANC, platelets, albumin, and creatinine clearance. Potential correlative covariates with *P* < 0.1 in univariate analyses were assessed in multivariate analysis by using a multiple linear regression model. All tests were 2-sided and a *P*-value of < 0.05 was considered to be statistically significant. All statistical analyses were performed using SPSS 18.0 (SPSS Inc., Chicago, IL).

## 3 Results

### 3.1 Characteristics of patients and Cmin distribution

Overall, 23 blood samples were obtained from 18 patients. IM Cmin was measured twice in 4 patients treated with 300 mg/d IM and 1 patient treated with 400 mg/d IM. The average age of the patients was 52 years (range 34–88 years). Eight patients (44.4%) are male. Primary tumor was located in the stomach in 11 patients (61.1%), small intestine in 6 patients (33.3%), and rectum in 1 patient (5.6%). Twelve patients underwent surgical resection of the primary tumor. All the patients were considered at high risk of recurrence according to modified NIH criteria and have been treated with IM 1 month after operation. Six patients have unresectable or metastatic GIST and have been taking IM since diagnosis. KIT exon 11 mutations were detected in 11 patients. The median follow-up was 13 months (7–31 months).

**Figure 1:**
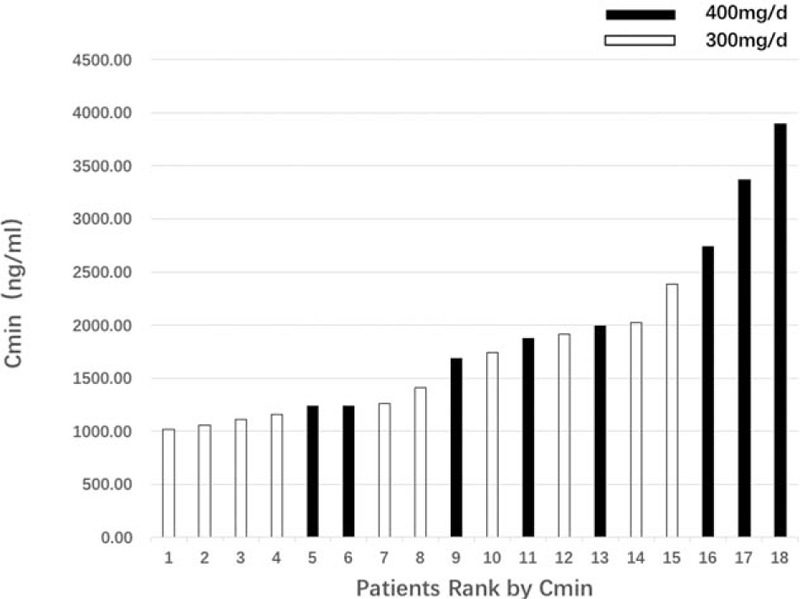
Distribution of imatinib trough concentration (Cmin) at steady-state for 300 mg/d and 400 mg/d. Cmin = trough concentration.

Distribution of IM Cmin at steady state for 300 mg/d and 400 mg/d are displayed in Fig. 1. IM Cmin in all 18 patients reached over 1000 ng/mL, and the mean Cmin was 1841 ng/mL (1018–3897 ng/mL). Distribution of IM Cmin had between sex and ages and primary sites of tumors (Table 1). In univariate analysis, we failed to reveal the association between IM Cmin and factors including BMI, hemoglobin level, WBC, creatinine clearance, or albumin level. The body surface area was the sole potential covariate for IM C min (coefficient: −0.22, *P* = 0.08). Meanwhile, the multivariate analysis was not performed (Table 2).

**Table 1:**
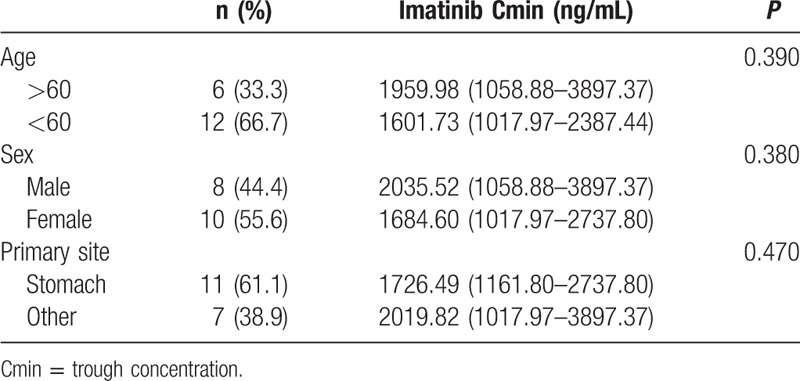
Distribution of imatinib in different age, sex, and primary site.

**Table 2:**
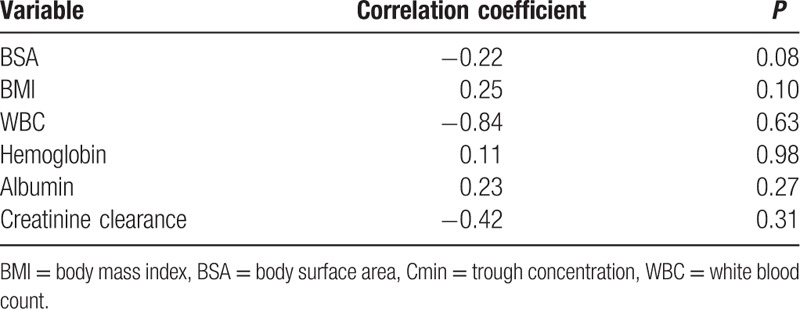
Univariate analysis of covariates for imatinib Cmin.

### 3.2 Efficacy of dose reduction

**Figure 2:**
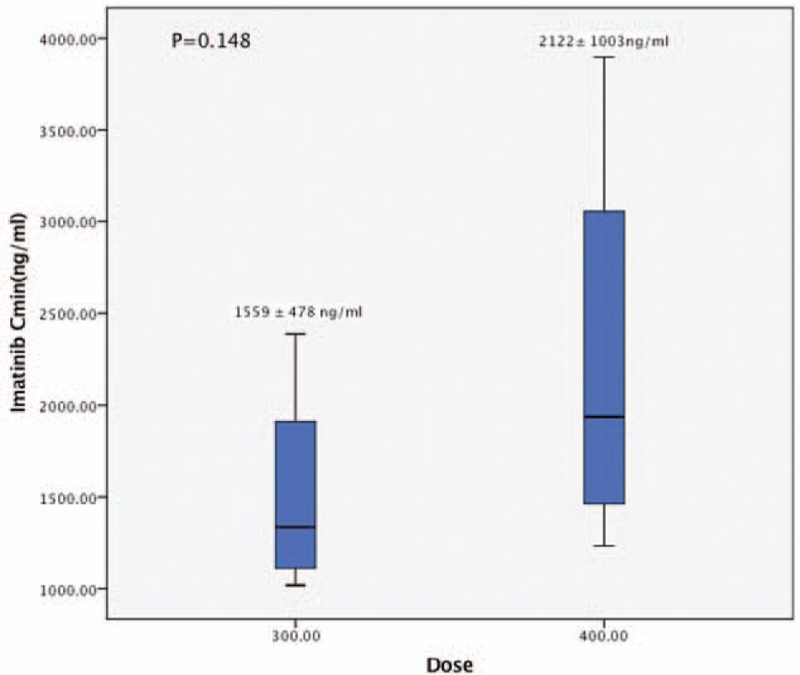
Imatinib plasma trough levels (Cmin) relative to daily dose. Cmin = trough concentration.

The mean of IM Cmin in patients receiving 300 mg daily dose was 1559±478 ng/mL (median 1412 ng/mL, range 1018–2387 ng/mL). The median time between dose reduction and PK tests was 8 months (4–16 months). In contrast to patients treated with 400 mg/d, the Cmin in patients receiving 300 mg/d decreased, but not significantly (2122±1003 ng/mL vs 1559±478 ng/mL, *P* = 0.148, Fig. 2).

In 12 patients who had undergone surgery, 4 had been taking 400 mg/d IM, and 8 patients were treated with 300 mg/d. The mean of IM Cmin was 2262±1009 ng/mL and 1596±496 ng/mL (*P* = 0.174). After a median follow up of 12.5 months (7–31 months), no recurrence was recorded in the groups.

In 6 patients who have unresectable or metastatic GIST, 5 were treated with 400 mg/d IM. The mean (±standard deviation) IM Cmin was 2011±1103 ng/mL, the median follow up was 14 months (7–18 months), and 4 PR and 1 SD were observed. Only 1 of the 6 patients received 300 mg/d IM, the Cmin was 1258 ng/mL, and the tumor size continued to decrease until the last follow up (13 months).

### 3.3 Correlation between IM exposure and toxicities

All the18 patients were initially treated with 400 mg/d. Under 400 mg/d dosage, 16 patients (88.9%) developed drug-related side effects; grade 3–4 toxicities were recorded in 4 patients (22.2%). The frequency of toxicity was lower (88.9% vs 44.4%, *P* = 0.023) in 9 patients treated with 300 mg/d IM (Table 3). Patients with grade 3–4 toxicities did not differ from patients with zero or mild toxicities in terms of IM Cmin (1875±173 vs 1834±896, *P* = 0.940). However, after dose reduction, all 9 patients exhibited improvement in the mitigation of the side effects at varying degrees: 4 patients had complete relief, and the other patients only exhibited grade 1 toxicity, and 1 patient only exhibited grade 3 rash and his IM level is 2026 ng/mL at 300 mg/d. The frequency and the degree of change after dose reduction for each individual patient are shown in detail in Table 4.

**Table 3:**
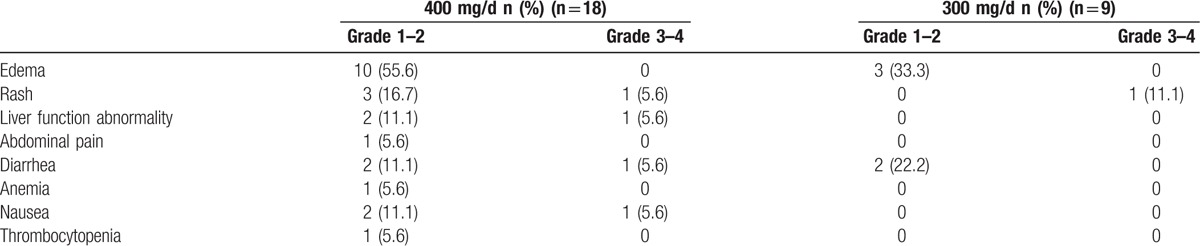
Adverse events at imatinib 400 mg/d vs 300 mg/d.

**Table 4:**
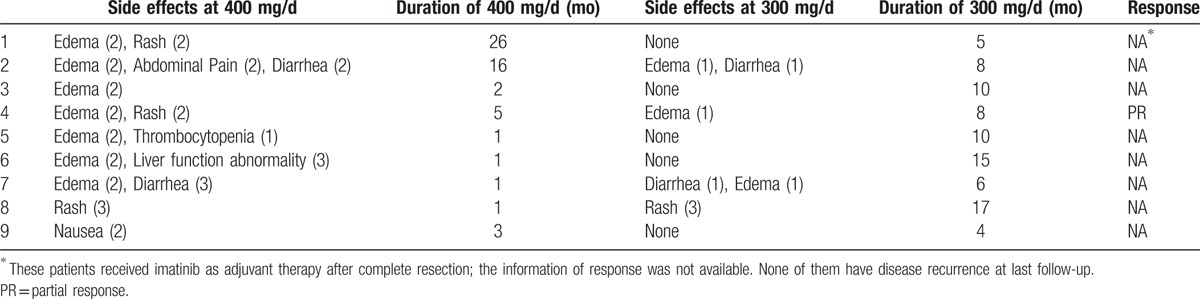
Side effects, dose of imatnib, and course of the treatment.

## 4 Discussion

This retrospective study showed that dose reduction of IM to 300 mg/d is feasible and effective in GIST patients who exhibit intolerance to standard dose of IM.

A standard dose of 400 mg/d IM has been gradually established by previous studies and has been recommended by all current clinical guidelines. In the phase I of the study, increased IM doses from 400 mg/d to 1000 mg/d resulted in severe toxicity; thus, IM 800 mg/d was found to be the maximum tolerated dose.^[13]^ Meanwhile, B2222 tails showed that IM at 400 mg/d and 600 mg/d induced desirable disease control in unresectable or metastatic GIST patients.^[3]^ Subsequently, EORTC62005 and S0033 trails assessed the efficiency of IM at 400 mg/d and 800 mg/d dose. Both studies reported the equivalent response rate and overall survival in these dose levels, but a higher dose of IM was observed to have a certain advantage in PFS. This evidence confirmed the efficiency and safety of IM at a 400 mg/d dose and also suggested that increasing the dose to 800 mg/d could regain disease control when patients progressed at the 400 mg/d dose.^[4,5]^

The increased dosage of IM improved the clinical response through increased plasma exposure. In the PK study based on B2222 trials, Demetri et al^[8]^ demonstrated that in patients with advanced GIST, IM Cmin values at steady-state (day 29 of treatment) were associated with clinical benefits. Patients with IM Cmin below 1100 ng/mL showed less clinical benefits. On the other hand, the higher level of Cmin was associated with increased frequency and severity of toxicities. Adverse events are the main cause of the IM interruption, which had been proven to result in the progression in GIST patients. According to previous studies, even with 400 mg daily dose, the incidence of mild adverse reaction can be over 90%, and grade 3–4 toxicities occurred in ∼30% of the patients,^[4,5]^ especially in Asian patients who were reported to be more vulnerable to IM toxicity at 400 mg/d.^[14]^ Dose reduction can be the most common solution to relieve toxicities, but the efficiency of low dose IM is unclear. In a recent case report, the IM dose was gradually reduced from 400 mg/d to 200 mg/d in Korea patients who suffered from severe rash, nausea, and vomiting. The gradual decrease reduced the size of the metastatic GIST and notably mitigated the adverse events.^[15]^ Meanwhile, Faber et al^[16]^ reported that lower dose IM can be effective in patients with chronic myeloid leukemia. Considering the high inter-individual pharmacokinetic variability of IM, these results suggest that a more individualized treatment is more appropriate than methods with fixed dosage of IM.

The primary objective of this study is to determine whether 300 mg/d dose IM can provide sufficient plasma exposure. The optimal threshold value of IM Cmin has yet been determined in patients with GIST. Demetri et al^[8]^ suggested that a Cmin of >1100 ng/mL may be a suitable threshold for better clinical outcomes in patients with advance GIST who are treated with IM 400 or 600 mg/d. In our study, IM plasma Cmin at 300 mg/d was measured in 9 patients. The mean IM Cmin was 1559±478 ng/mL (1017.97 ng/mL to 2387.44 ng/mL), which was compared with patients who were treated with 400 mg/d IM. IM plasma Cmin has been found to be affected by several clinical factors,^[17,18]^ including albumin, white blood cells, granulocytes, hemoglobin, creatinine clearance, and previous major gastrectomy. However, in this study, we failed to identify the association of IM Cmin with any of these factors, probably because of the small sample size. Consistent with previous reports, there is a large inter-patient variability in imatinib blood levels in the present study, which could be explained by genetic polymorphisms of cytochrome P450 enzymes involved in IM metabolism. Previous population PK study of patients with GIST suggested that IM plasma Cmin decreases with prolonged administration. However, the information on Cmin at the initial stages of the therapy is not available in the present study; thus, the association between the changes in Cmin and long-term therapy was not determined.

IM in an adjuvant setting after complete resection of GIST have been reported to be effective in the ACOSOG Z9001 and the SSGXVIII/AIO study.^[19,20]^ However, there have been no data available on the effectiveness of reduced dose of IM mesylate as an adjuvant therapy for GIST. Most of the patients in the patient study who were treated with 300 mg/d IM had previously undergone complete surgical resection and the median duration of low-dose postoperative IM treatment was 8 months (4–14 months). None of these patients developed recurrence after a median follow-up time of 12.5 months (7–31 months) after surgery. The recommended course of postoperative IM is at least 36 months,^[11]^ and additional follow-ups are ongoing to confirm the effectiveness of 300 mg/d IM in this setting. Clinical trials tend to prolong the postoperative IM duration because of the findings that stopping adjuvant therapy is followed by relapse in most patients,^[21]^ but no evidence has shown that higher dose in postoperative therapy can prevent relapse after cessation of treatment. If lower dose IM is as effective as the standard dose in adjuvant setting, it will not only alleviate the side effects but also reduce treatment cost. One patient with multiple liver metastatic GIST in this series received 300 mg/d IM The size of metastasis of that patient continued to decrease after 13 months of treatment. Kinase mutational status can be used as a predictor of response to IM, as patients carrying KIT exon 11 mutations can achieve disease control at higher certainty than those with KIT exon 9 mutations or wild type on 400 mg/d IM.^[4,5]^ However, information on KIT and PDGFRA mutation status were only available in 11 patients in the present study who had KIT exon 11 mutation. Thus, we cannot analyze the influence of different mutations on the effects of low dose IM.

The main purpose of dose reduction is to manage toxicities. Tolerance for IM treatment is notably variable in different patients, particularly, patients treated with higher dose of IM and have higher plasma concentration do not necessarily suffer the worse side effects compared to those who were treated with lower dose and lower plasma concentration. However, the severity and frequency of toxicities significantly decreased in each patient after dose reduction. The side effect of IM has been reported to have improved with prolonged therapy, but in this series of patients, we recorded severe eyelid edema which lasted for more than 2 years, repeated diarrhea for 16 months, nausea for 3 months, and grade 2 rash in 1 patient after 4 months of treatment at 400 mg/d. These long-term side effects compromised the quality of life of the patients and could be the reason for the poor compliance in treatment. Similar situation has been showed in CML patients,^[22]^ wherein up to 30% of the patients experienced moderate to severe side effects for over a year. Lowering the dose based on the comprehensive discussion with a multiple disciplinary team and on the agreement between the patient and physician may be a solution to the long-term side effects and poor compliance in some patients. Monitoring of IM Cmin is essential in patients who received lower dose therapy.

We still cannot completely conclude that 300 mg/d can be a routine dose in all the GIST patients suffering from drug-related side effects because of limitation imposed by the retrospective design and small sample size. Nevertheless, our findings strongly support further and prospective studies to assess the efficiency and the safety of lower dose IM, especially in the adjuvant setting.
